# Cerebellar direct current stimulation modulates hand blink reflex: implications for defensive behavior in humans

**DOI:** 10.14814/phy2.13471

**Published:** 2018-05-15

**Authors:** Tommaso Bocci, Roberta Ferrucci, Davide Barloscio, Laura Parenti, Francesca Cortese, Alberto Priori, Ferdinando Sartucci

**Affiliations:** ^1^ Department of Clinical and Experimental Medicine Cisanello Neurology Unit Pisa University Medical School Pisa Italy; ^2^ Department of Medical‐Surgical Pathophysiology and Transplants University of Milan Milan Italy; ^3^ Clinical Center for Neurotechnology Neurostimulation and Movement Disorders Fondazione IRCCS “Ca' Granda” Ospedale Maggiore di Milano Milan Italy; ^4^ Department of Neurological Sciences University of Milan Fondazione IRCCS Ospedale Maggiore Policlinico Milan Italy; ^5^ Department of Medical‐Surgical Sciences and Biotechonologies Sapienza University of Rome Rome Italy; ^6^ Neuroscience Institute National Research Council Pisa Italy

**Keywords:** Cerebellar direct current stimulation, cerebellum, defensive behavior, hand blink reflex, peripersonal space, tDCS

## Abstract

The cerebellum is involved in a wide number of integrative functions. We evaluated the role of cerebellum in peripersonal defensive behavior, as assessed by the so‐called hand blink reflex (HBR), modulating cerebellar activity with transcranial direct current stimulation (tDCS). Healthy subjects underwent cerebellar (sham, anodal, and cathodal tcDCS) and motor cortex tDCS (anodal or cathodal; 20′, 2 mA). For the recording of HBR, electrical stimuli were delivered using a surface bipolar electrode placed on the median nerve at the wrist and EMG activity recorded from the orbicularis oculi muscle bilaterally. Depending on the hand position respective to the face, HBR was assessed in four different conditions: “hand‐far,” “hand‐near” (eyes open), “side hand,” and “hand‐patched” (eyes closed). While sham and cathodal cerebellar stimulation had no significant effect, anodal tcDCS dramatically dampened the magnitude of the HBR, as measured by the area under the curve (AUC), in the “hand‐patched” and “side hand” conditions only, for ipsilateral (*F*_(4,171)_ = 15.08, *P* < 0.0001; *F*_(4,171)_ = 8.95, *P* < 0.0001) as well as contralateral recordings (*F*_(4,171)_ = 17.96, *P* < 0.0001); *F*_4,171)_ = 5.35, *P* = 0.0004). Cerebellar polarization did not modify AUC in the “hand‐far” and “hand‐near” sessions. tDCS applied over the motor area did not affect HBR. These results seem to support a role of the cerebellum in the defensive responses within the peripersonal space surrounding the face, thus suggesting a possible cerebellar involvement in visual‐independent defensive behavior.

## Introduction

In the Sherringtonian model reflex responses provide a rapid and stereotyped first line of defense, by adequately reacting to aversive stimuli and optimizing the chances of survival (Sherrington 1906). This model has been recently improved (Castegnetti et al. 2016; Khemka et al. 2017), in accordance with Bayesian theories posing that the brain uses probabilistic inference and stores forward models and prior probabilities to compute optimal behavior (Bach 2015).

Psychophysical experiments on the attentional deficits of brain‐damaged humans have corroborated the hypothesis of specialized attentional mechanisms for the defensive peripersonal space (Ladavas et al. 1998; Pavani and Castiello 2004). A specific network, ranging from the polysensory zone (PZ) in the precentral gyrus to the ventral intraparietal (VIP) area, has been suggested to encode the defensive behavior within the space immediately around the body (Graziano et al. 1997, 1999; Duhamel et al. 1998; Macaluso and Maravita 2010). Neurons in VIP and PZ areas are multimodal, responding to tactile, visual, and auditory stimuli (Rizzolatti et al. 1981; Schlack et al. 2005): whereas frontal areas (PZ) improve motor output, parietal cortex (VIP) likely emphasizes sensory processing, attention, and planning. At a subcortical level, cross‐modal summation of multisensory stimuli likely occurs in the ventral spinal cord, where the integration between vestibulospinal and reticulospinal pathways mediates the startle reflex and facilitates the subsequent behavioral responses needed to escape from predation and blows (Yeomans et al. 2002).

In this scenario, the role of the cerebellum in the defensive behavior within the peripersonal space has not been clearly defined so far. The cerebellum is involved in a wide number of integrative functions, ranging from working memory and associative learning to motor control (Stoodley and Schmahmann 2009; Strick et al. 2009; Balsters et al. 2013); it also plays a role in the sensory‐motor integration aimed at antinociceptive behavior, as well as in salience‐related affective and behavioral responses to nociceptive stimulation (Bingel et al. 2002; Strigo et al. 2003; Bocci et al. 2015, 2016).

Here, we evaluated the role of cerebellum in defensive responses, as assessed with the so‐called hand blink reflex (HBR), by noninvasively modulating cerebellar activity with transcranial direct current stimulation (tDCS). Cerebellar tDCS is a novel, safe, and effective neurostimulation technique for noninvasive and polarity‐dependent modulation of cerebellar excitability, with short‐ and long‐term effects likely arising from the depolarization of Purkinje and Golgi cells, respectively (Priori et al. 2014; Ferrucci et al. 2016). Despite some interindividual differences, recent modeling studies have revealed that during cerebellar tDCS the current spread to other structures outside the cerebellum is negligible and unlike to produce functional effects (Parazzini et al. 2014; Fiocchi et al. 2016).

HBR represents a nonstereotyped defensive behavior; it is mediated at brainstem level, likely undergoing tonic top–down modulation from higher order cortical areas responsible for encoding the location of somatosensory stimuli in external space coordinates (Sambo et al. 2012a,b; Bufacchi et al. 2016; Fossataro et al. 2016). HBR is enhanced when the stimulated hand is located inside the peripersonal space surrounding the face, irrespective of whether the eyes are closed or not (Sambo et al. 2012b), in contrast with previous studies that have shown that vision of the body is crucial for proprioceptive localization (van Beers et al. 1999) and attentional selection (Sambo et al. 2009).

## Materials and Methods

### Subjects

Twenty right‐handed healthy volunteers (ten women; mean age ± SD: 25.2 ± 5.4 year), with no history of neurological disorders, were enrolled. No subject had been under medication in the month preceding the experimental session, which was scheduled at least 48 h after the last alcohol and caffeine consumption. Written informed consent was obtained from all participants before enrollment in the study, which was approved by the local ethical Committee and followed the tenets of the Declaration of Helsinki.

### Study protocol

Each volunteer underwent cerebellar (sham, anodal, and cathodal) and motor cortex (anodal or cathodal) tDCS (total of five session per subject); sessions were separated by at least 1 week to avoid possible confounding after‐effects. During each session, we evaluated HBR area at baseline and immediately after cerebellar, or M1, polarization; HBR was recorded bilaterally from the orbicularis oculi muscle, in four different experimental conditions (“hand‐far,” “hand‐near,” “side hand,” and “hand‐patched”).

### Stimulation setting

Electrical stimuli were delivered using a surface bipolar electrode placed on the median nerve at the wrist. The stimulator was attached on the participants’ wrist with a rubber strap before the beginning of the recording, thus ensuring constant pressure across experimental conditions.

In accordance with the existing literature, stimulus intensity was adjusted, in each participant, to elicit a clear HBR in three consecutive trials (24–55 mA, mean of 38.9 mA), with a duration of 200 *μ*sec and the interval of about 30 sec (Sambo et al. 2012a,b). Although there could be some degree of variability as expected for a reflex mediated by a polysynaptic circuit in the reticular formation, a constant intertrial interval of 30 sec reduces habituation over time (Cruccu et al. 2006). In order to avoid interference from the return electrode placed over the contralateral shoulder, the left wrist was always the one to be stimulated.

### Hand blink reflex recording

Participants seated in a comfortable chair. EMG activity was recorded from the orbicularis oculi muscle, bilaterally, using pairs of surface electrodes with the active electrode over the mid‐lower eyelid and the reference electrode a few centimeters laterally to the outer canthus. Signals were amplified and digitized at a sampling rate of 8192 Hz (ISA 1004, Micromed^®^, Treviso, Italy), and stored for offline analysis. A facial response was considered positive when a burst of EMG activity, with an amplitude >50 *μ*V and a duration >10 msec, appeared consistently at a latency compatible with a reflex response earlier than a voluntary reaction (Valls‐Sole et al. 1997). Although none of the participants has reported pain, at the high stimulation intensities used to elicit the HBR we cannot exclude that populations of fibers other than A*β* were recruited; the onset–offset latencies of the HBR were set at 45 and 100 msec, respectively (Sambo et al. 2012a,b), as these temporal limits rule out any contribution of A*δ* and C afferents (Moraux et al., 2010).

To investigate the effect of hand position on the HBR, before (T_0_) and after (T_1_, T_2_) tcDCS, we used four different experimental conditions by stimulating the left side (Sambo et al. 2012a,b). In the first (the “hand‐far” condition), participants were sitting with their forearm resting on a table, at ∼120° with respect to the arm; such posture resulted in the wrist being at a distance of ∼60 cm from the ipsilateral side of their face. The palm of the hand and the fingers were below the lower limit of the visual field.

In the second type of recording (“hand‐near”), participants were sitting with their arm resting on a table, the forearm at ∼75° with respect to the arm, and the wrist at a distance of ∼4 cm from the ipsilateral side of their face. These distances corresponded to hand positions clearly outside (“far” condition) and inside (“near” condition) the peripersonal space of the face (Farne et al. 2005).

In the third type (“side hand” condition) we recorded the HBR while the position of the head was kept constant and the arm was rotated sideways by 90° respect to “near” condition.

To investigate the contribution of the vision of the stimulated hand in “near” condition, in the fourth type we recorded the HBR while they kept their eyes covered by surgical patches.

During all the experimental conditions the fingers, the palm of the hand, the wrist, or any other part of the upper limb were never touching the face or the head. The hand not undergoing the postural manipulation was never stimulated, and the arm was held along the body throughout the duration of the experiment.

A total of 32 electrical stimuli were delivered to the median nerve, in separate blocks. In each block, 8 stimuli were delivered in the far condition, 8 in the near condition, 8 in the near‐side condition and 8 in the near condition without vision. The order of the four experimental conditions (hand‐far, hand‐near, hand‐side, hand‐patched) was randomized among participants and stimulation conditions.

The stimulation procedure did not produce any sound, to mask any auditory cue throughout the experiment.

### Transcranial direct current stimulation (tDCS)

After the preliminary recording the subjects were submitted to tDCS applied either over the cerebellum (transcutaneous cerebellar direct current stimulation, tcDCS) or the primary motor area, using a battery‐driven constant current stimulator (HDCStim, Newronika, Italy) and a pair of electrodes in two saline‐soaked synthetic sponges with a surface area of 35 cm^2^ (7 × 5 cm). Direct current was transcranially applied for 20 min with an intensity of 2.0 mA, and constant current flow was measured by an ampere meter (current density ≈ 0.08 mA/cm^2^). These values are similar to those previously reported for cerebellar stimulation (Ferrucci et al. 2008, 2013), are considered to be safe (Iyer et al. 2005) and are far below the threshold for tissue damage (Nitsche et al. 2003b). Apart from occasional and short‐lasting tingling and burning sensations below the electrodes, direct current stimulation strength remained below the sensory threshold throughout the experimental session. At the offset of tDCS, the current was decreased in a ramp‐like manner, a method shown to achieve a good level of blinding among sessions (Gandiga et al. 2006; Galea et al. 2009).

For anodal cerebellar stimulation, the anode was centered on the median line 2 cm below the inion, with its lateral borders about 1 cm medially to the mastoid apophysis, and the cathode over the right shoulder (Ferrucci et al. 2012, 2013; Bocci et al. 2015). For cathodal polarization, the current flow was reversed. We have adopted a bilateral stimulation as previous studies have shown that varying the position of the active electrode with ±1 cm only induced a small change in the field amplitude distributions (Parazzini et al. 2014).

For M1 stimulation, the active electrode was placed over the right motor hotspot (C4 scalp positions of the International EEG 10/20 system), identified by single pulses of TMS delivered at a slightly suprathreshold stimulus intensity to elicit responses on the first dorsal interosseus muscle. TMS was delivered using a 70‐mm loop‐diameter figure‐of‐8 coil (2.2 T maximum field output; Magstim Company, Dyfed, UK). The return electrode was placed on the skin overlying the contralateral supraorbital region (Nitsche and Paulus 2000; Ardolino et al. 2005; Galea et al. 2011).

For a sham tDCS, the current was turned on only for 5 sec at the beginning of the sham session and then it was turned off in a ramp‐shaped fashion, which induces initial skin sensations indistinguishable from real tDCS.

At experimental debriefing, subjects were not able to discriminate between the applied anodal and cathodal.

Participants were blinded to the tcDCS polarity; anodal, cathodal, and sham tcDCS stimulations were administered in three different sessions and separated by at least 1 week to avoid possible carry‐over effects. The order of interventions was randomized and balanced across subjects.

### Statistical analysis

EMG signals from each participant were high‐pass filtered (55 Hz), full‐wave rectified, and averaged separately for the each condition, at the ipsilateral and contralateral recording sides (Sambo et al. 2012a,b). Parametric analyses were used, as all datasets successfully passed the Shapiro–Wilk test for normality (*P* > 0.05). A one‐way ANOVA was used to compare baseline values for each subject among sham, anodal, and cathodal condition. In each participant, we measured the area under the curve (AUC) of the HBR for each experimental condition and recording side.

Electrophysiological measures were normalized to baseline before entering the analysis (according to the formula (T_1_‐T_0_)/T_0_*100 + 100); a two‐way repeated‐measures (RM) ANOVA was performed, with “stimulation” (three levels: anodal, cathodal, and sham) and “time” (three levels: T_0_, T_1_, and T_2_) as experimental factors, followed by Holm–Sidak post hoc method a two‐way RM ANOVA, with “stimulation” (two levels: anodal and cathodal) and “time” (three levels: T_0_, T_1_, and T_2_) as factors, was also run to assess possible changes in HBR area following tDCS applied over M1.

Statistical significance was set at *P* < 0.05. Data were analyzed using SPSS v. 21.0 for Windows (SPSS Inc.).

## Results

### Hand‐far and hand‐near conditions

**Figure 1:**
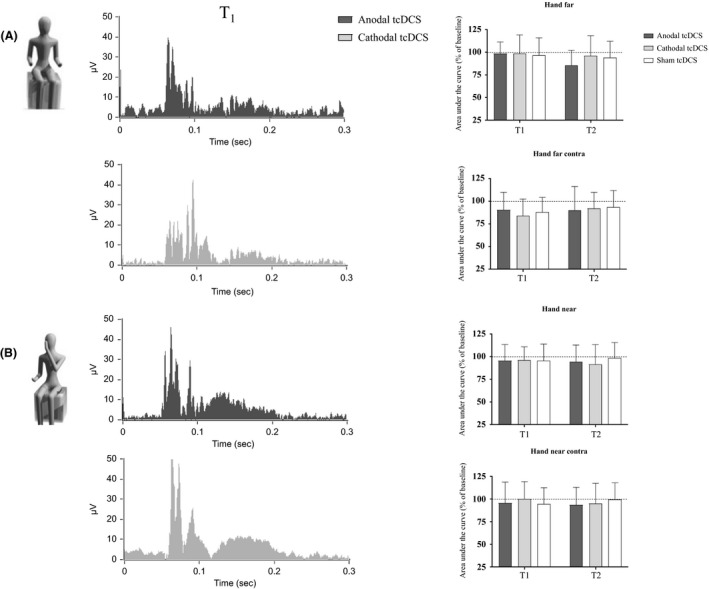
“Hand‐far” (A) and “hand‐near” (B). In the first two experimental conditions, no significant modification of AUC appeared following either anodal or cathodal cerebellar polarization. At the left: ipsilateral traces at T1 were grand‐averaged and rectified (black: anodal tcDCS; gray: cathodal tcDCS). At the right: histograms showing trend over time of AUC following anodal, cathodal, and sham stimulation, both for ipsilateral (at the top) and contralateral (bottom) recordings (gray: cathodal tcDCS; dark gray: anodal tcDCS; white: sham tcDCS).

Changes in AUC over time are reported in Table 1 and Figure 1. Baseline values did not change among different sessions (*P* > 0.3 for all the experimental sessions, one‐way ANOVA with “stimulation” as factor). For ipsilateral recordings, tcDCS did not modify AUC both in hand‐far (*F*_(4,171)_ = 1.04, *P* = 0.39, two‐way repeated‐measures ANOVA, with “stimulation” and “time” as factors) and hand‐near conditions (*F*_(4,171)_ = 0.37, *P* = 0.83). By analogy, for contralateral recordings no significant change was found following the completion of cerebellar polarization (hand‐far; *F*_(4,171)_ = 0.45, *P* = 0.77; hand‐near: *F*_(4,171)_ = 0.56, *P* = 0.69). Table 1 shows *P* values when different time points for the same polarization were compared. Latencies of single traces were computed for each subject and did not change among different experimental conditions (*P* > 0.1), ranging from 53.2 to 65.1 msec in hand far condition, from 50.4 to 62.9 msec for hand‐near sessions.

**Table 1:** Changes in AUC induced by cerebellar tDCS (tcDSC)

		Anodal		Cathodal		Sham	
		Ipsilateral	Contralateral	Ipsilateral	Contralateral	Ipsilateral	Contralateral
Hand‐far	*T0*	4223.9 ± 1142	3573.4 ± 923	4143.3 ± 926	3273.5 ± 757	3996.2 ± 742	3210.3 ± 667
	*T1*	4138.8 ± 984	3703.7 ± 1026	3987.6 ± 971	2674.6 ± 616	3839.3 ± 960	2765.7 ± 535
	*T2*	3543.9 ± 846	3103.1 ± 911	3902.4 ± 1014	2962.8 ± 783	3754.2 ± 947	2962.8 ± 671
Hand‐near	*T0*	4449.5 ± 1196	3499.1 ± 842	4006.3 ± 808	3229.1 ± 527	4043.1 ± 583	3247.2 ± 632
	*T1*	4200.2 ± 1116	3309.6 ± 928	3791.3 ± 562	3187.8 ± 537	3808.8 ± 577	3053.2 ± 685
	*T2*	4118.9 ± 1007	3202.2 ± 5809	3605.5 ± 823	3029.4 ± 636	3913.3 ± 465	3170.1 ± 502
Side‐Hand	*T0*	3956.8 ± 1582	2978.2 ± 821	3809.3 ± 751	2797.2 ± 615	4096.0 ± 897	3182.6 ± 898
	*T1*	2224.5 ± 742	1804.9 ± 890	3343.6 ± 686	2775.6 ± 947	3610.3 ± 847	2923.1 ± 1087
	*T2*	2412.3 ± 1233	2012.1 ± 610	3357.9 ± 859	2753.3 ± 707	3587.1 ± 961	2879.9 ± 905
Hand‐patched	*T0*	4053.1 ± 1313	3425.3 ± 1129	3701.1 ± 772	2922.9 ± 903	3852.1 ± 809	2909.4 ± 773
	*T1*	2388.0 ± 667	1825.9 ± 984	3609.4 ± 699	3008.6 ± 798	3712.6 ± 754	2770.1 ± 683
	*T2*	2571.2 ± 834	2023.7 ± 667	3584.4 ± 932	2971.3 ± 914	3631.4 ± 908	2751.8 ± 823

Effects of cerebellar polarization on the area under the curve (AUC). Values are expressed as *μ*Vms. Notably, significant effects were found only in the “hand‐patched” and “side‐hand” conditions after the completion of anodal polarization. Relative *P*‐values are reported in the text.

### Side‐hand and hand‐patched

**Figure 2:**
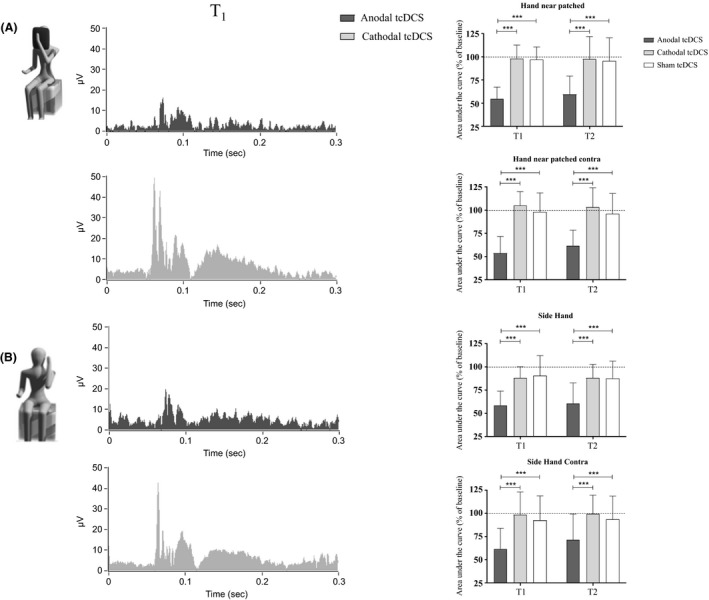
“Hand‐patched” (A) and “side‐hand” (B). Anodal stimulation significantly dampened AUC compared both with sham and cathodal polarization (**P < 0.01; ***P < 0.0001). At the left: traces at T1 were grand‐averaged and rectified (black: anodal tcDCS; gray: cathodal tcDCS). At the right: histograms showing AUC changes following anodal, cathodal, and sham stimulation (gray: cathodal tcDCS; dark gray: anodal tcDCS; white: sham tcDCS).

Remarkably, significant differences were found when the visual feedback was removed (Fig. 2). tcDCS changed AUC both for side‐hand and hand‐patched conditions, for ipsilateral (side hand; *F*_(4,171)_ = 8.95, *P* < 0.0001; hand‐patched: *F*_(4,171)_ = 15.08, *P* < 0.0001, two‐way RM‐ANOVA, with “stimulation” and “time” as factors), as well as contralateral recordings (*F*_(4,171)_ = 5.35, *P* = 0.0004; *F*_(4,171)_ = 17.96, *P* < 0.0001).

When analyzed separately, whereas cathodal polarization had no significant effect (side‐hand: *F*_(2,114)_ = 0.1, *P* = 0.89; hand‐patched: *F*_(2,114)_ = 0.05, *P* = 0.9), anodal tsDCS reduced AUC compared with sham condition (side‐hand: *F*_(2,114)_ = 11.4, *P* < 0.0001; hand‐patched: *F*_(2,114)_ = 22.9, *P* < 0.0001, two‐way repeated measures ANOVA, “stimulation” and “time” as factors, followed by Holm–Sidak test). Also for contralateral recordings, whereas cathodal stimulation left AUC unchanged (side‐hand: *F*_(2,114)_ = 0.3, *P* = 0.74; hand‐patched: *F*_(2,114)_ = 0.6, *P* = 0.55), anodal tcDCS decreased it with respect to sham condition (side‐hand: *F*_(2,114)_ = 5.93, *P* = 0.0035; hand‐patched: *F*_(2,114)_ = 21.8, *P* < 0.0001). Figure 1 shows p values when different time points for the same polarization were compared.

Latencies of single traces were computed for each subject and did not change among different experimental conditions (*P* > 0.1), ranging from 48.6 to 58.7 msec in “hand‐near patched” condition, from 46.3 to 58.2 msec for “side‐hand” sessions.

### Stimulation of the primary motor area (M1)

**Figure 3:**
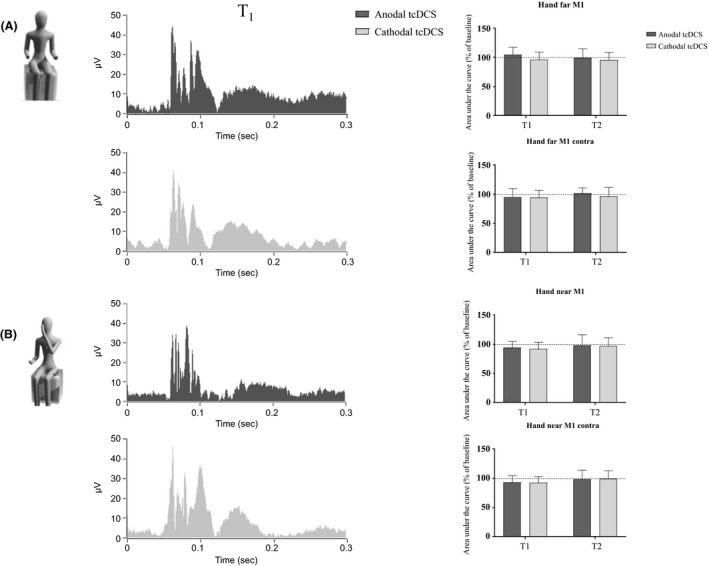
Stimulation of left M1. In the first two experimental conditions (“hand‐far”, A, and “hand‐near”, B), no significant modification of AUC appeared following either anodal or cathodal tDCS applied over M1 (gray: cathodal tDCS; black: anodal tDCS). At the right: histograms showing trend over time of AUC following anodal and cathodal stimulation, both for ipsilateral (at the top) and contralateral (bottom) recordings (gray: cathodal tDCS; dark gray: anodal tDCS).

Hand‐far and hand‐near. Changes in AUC over time are reported in Table 2 and Figure 3. When a two‐way ANOVA was run to evaluate the effects of tDCS over M1, no change was found both for ipsilateral (hand‐far: *F*_(2,114)_ = 0.62, *P* = 0.54; hand‐near: *F*_(2,114)_ = 0.1, *P* = 0.9, with “time” and “stimulation” as factors) and contralateral recordings (hand‐far: *F*_(2,114)_ = 0.5, *P* = 0.61; hand‐near: *F*_(2,114)_ = 0.03, *P* = 0.95; see Table 2 and Figure 3).

**Table 2:** Changes in AUC induced by motor cortex tDCS (M1 tDSC)

		Anodal		Cathodal	
		Ipsilateral	Contralateral	Ipsilateral	Contralateral
Hand‐far	*T0*	4137.3 ± 541.8	3555.9 ± 387.5	4198.9 ± 485.3	3477.3 ± 385.8
	*T1*	4270.8 ± 441.6	3372.9 ± 565.3	4038.3 ± 676.2	3271.1 ± 574.8
	*T2*	4090.7 ± 614.1	3596.4 ± 394.1	4011.9 ± 733.8	3328.0 ± 435.4
Hand‐near	*T0*	4691.1 ± 464.7	3375.5 ± 510.0	4316.5 ± 500.7	3545.1 ± 427.8
	*T1*	4287.6 ± 425.9	3118.7 ± 411.9	3946.9 ± 560.7	3274.6 ± 564.7
	*T2*	4554.9 ± 762.8	3320.1 ± 689.4	4178.1 ± 599.6	3526.2 ± 617.6
Side‐Hand	*T0*	3473.7 ± 449.6	3232.6 ± 734.5	3535.2 ± 588.9	3088.4 ± 762.7
	*T1*	3418.2 ± 321.9	2913.5 ± 672.4	3174.2 ± 707.1	3055.0 ± 414.4
	*T2*	3546.9 ± 538.6	3033.7 ± 722.8	3379.8 ± 668.4	2869.5 ± 713.6
Hand‐near patched	*T0*	3866.8 ± 692.1	3127.3 ± 430.0	4174.4 ± 500.9	2902.7 ± 524.3
	*T1*	3452.9 ± 634.2	2840.7 ± 679.7	3927.2 ± 536.3	2667.1 ± 425.1
	*T2*	3623.0 ± 540.1	2922.4 ± 525.3	3953.7 ± 637.4	2876.0 ± 731.2

Effects of M1 polarization (values are expressed as *μ*Vms). When the left primary motor cortex was stimulated, tDCS left HBR area unchanged following either anodal or cathodal stimulation. Of note, different from cerebellar tDCS, no significant effect was found in the “hand‐patched” and “side‐hand” conditions.

**Figure 4:**
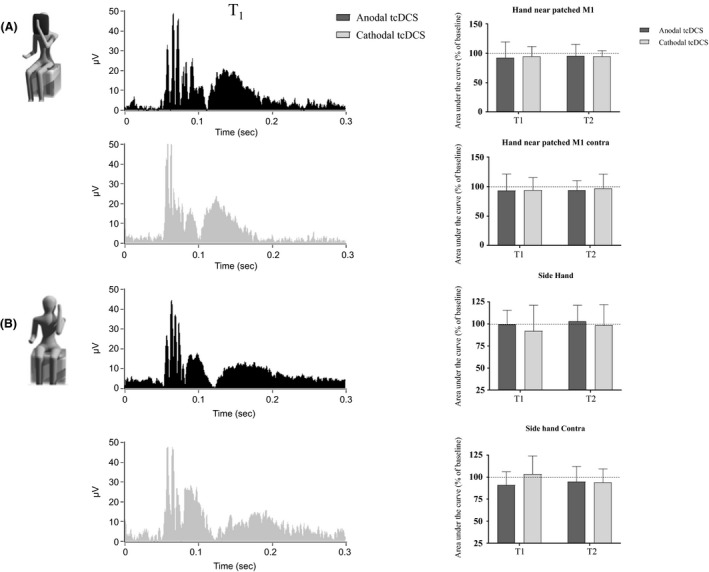
Stimulation of left M1 (“hand‐patched”, A, and “side‐hand”, B). Different from cerebellar stimulation, both anodal and cathodal polarization left HBR area unchanged compared with baseline values. At the left: traces at T1 were grand‐averaged and rectified (gray: cathodal tDCS; black: anodal tDCS). At the right: histograms showing AUC changes following anodal and cathodal stimulation (gray: cathodal tcDCS; dark gray: anodal tDCS).

Side‐hand and hand‐patched different from cerebellar polarization, no change was found for ipsilateral (side‐hand: *F*_(2,114)_ = 0.8, *P* = 0.45; hand‐near patched: *F*_(2,114)_ = 0.23, *P* = 0.79, “stimulation” and “time” as factors; Fig. 4) and contralateral recordings (side‐hand: *F*_(2,114)_ = 0.63, *P* = 0.53; hand‐near patched: *F*_(2,114)_ = 0.06, *P* = 0.91; see Table 2 and Fig. 4). Remarkably, when changes induced by anodal polarization were compared between M1 and cerebellar tDCS, a significant site effect was found, with anodal tcDCS dramatically dampening AUC both for ipsilateral (side‐hand: *P* = 0.0004; hand‐near patched: *P* = 0.0013, two‐way RM ANOVA with “site” as factor) and contralateral recordings (side‐hand: *P* < 0.0001; hand‐near patched: *P* = 0.0001). When analyzed at different time intervals, baseline values were similar (*P* > 0.05) with consistent differences appearing at T1 and T2, for ipsilateral (side‐hand: *P* = 0.001 and *P* = 0.0018; hand‐near patched: *P* = 0.0008 and *P* = 0.0007) and contralateral recordings (side‐hand: *P* < 0.0001 and *P* = 0.0004; hand‐near patched: *P* = 0.0004 and *P* = 0.002, T1 and T2, respectively, Holm–Sidak post hoc method).

## Discussion

Cerebellar tDCS is able to modulate HBR when the stimulated hand is located inside the peripersonal space surrounding the face, thus suggesting a possible cerebellar involvement in the defensive peripersonal behavior in humans: whereas cathodal and sham stimulation have no significant effect, anodal polarization reduces AUC. Curiously, as anodal tDCS modifies reflex responses in the “near‐side” and “eyes patched” conditions, cerebellum seems to interfere with defensive behavior selectively when the visual feedback is missing.

As previously reported by our group (Ferrucci et al. 2012), cerebellum likely belongs to a widespread network that mediates reactions stronger to negative external stimuli than to positive ones, a phenomenon referred as “negative bias” (Fox et al. 2000; Morewedge 2009): by allowing individuals to adapt to the environment, it ultimately favors survival of species. Present results fit also with lesional models suggesting that plasticity subserving eyeblink conditioning, responsible for motor learning, selectively occurs in the cerebellum (Bracha et al. 1999, 2001; Timmann et al. 2000; Galea et al. 2011).

Our results prompt to further questions: (1) what is the putative role of the cerebellum in defensive peripersonal behavior? (2) which are neural mechanisms underlying HBR genesis?

The cerebellum and its related brainstem nuclei are critically involved in the control and production of the classically conditioned eyeblink response and may contain essential long‐term neuronal changes which serves to encode this learned response (McCormick et al. 1983); a cerebellar role in the retention and storage of conditioned responses, as assessed by visual threat eyeblink responses, has been recently confirmed (Thieme et al. 2013). Overall, the cerebellum is engaged in learning of unspecific aversive reactions, also outside the peripersonal space (Frings et al. 2006), and cerebellar dysfunction may lead to impaired short‐term and long‐term habituation of the startle response (Maschke et al. 2000; Lafo et al. 2017).

However, beyond the traditional view of a selective involvement in the visuo‐motor integration, some studies have suggested a more sophisticated role of the cerebellum. In particular, both the right inferior parietal lobe and the left posterior cerebellum are likely engaged in decoupling visuo‐motor and multisensorial interactions, thus overcoming our default ability to functionally integrate arm and eye movements (Synofzik et al. 2008; Izawa et al. 2012; Gorbet and Sergio 2016). Moreover, cerebellum is not necessary for visually driven recalibration of hand proprioception (Henriques et al. 2014), as proved by the preservation of visual‐propriocetive discrepancy signal in cerebellar patients (Synofzik et al. 2008; Henriques and Cressman 2012; Izawa et al. 2012). Similarly, our findings seem to suggest a role of the cerebellum in peripersonal behavior when the visual feedback is lost; as tDCS does not interfere with HBR when the hand is close to the face at eyes open, it's unlikely that its effects merely depend on cognitive expectations.

Regarding its genesis, HBR probably originates at brainstem level undergoing tonic top‐down modulation from higher order cortical regions (Sambo et al. 2012a,b; Sambo and Iannetti 2013); cerebellum possibly integrates these networks and coworks with cerebral cortex in its regulation. As it bilaterally interferes with reflex responses when the visual feedback is lost, cerebellum may in part exert its role alone, independently from any cortical control. Cerebellum could not only integrate nonmotor functions, but also disentangle different channels carrying multisensory information (Henriques et al. 2014). This peculiar and selective role could be further confirmed by the results obtained with M1 tDCS; different from cerebellar polarization, the effect on M1 was not statistically relevant and appeared with and without the visual feedback.

Finally, the fact that anodal, but not cathodal tcDCS, affects the HBR is intriguing. Anodal and cathodal stimulation likely exert effects through different, rather than simply specular, mechanisms of action on different cellular and molecular targets, in accordance with those reported for the cerebral cortex (Stagg et al. 2009). The polarity of cerebellar tDCS after‐effects may also depend on the montage used (van Dun et al. 2016, 2017) and the function explored, as different functions rely on different cerebellar areas with variable neural substrates and axonal orientation to the electrical field (Ferrucci et al. 2016). Overall, our results seem to confirm previous data showing that excitatory anodal tcDCS enhances online acquisition of new motor skills (Cantarero et al. 2015), whereas cathodal stimulation does not affect motor behavior (Nitsche et al. 2003a; Reis et al. 2009).

### Limitations and alternative explanations

Direct current stimulation applied near the mastoid process may influence the firing behavior of primary vestibular afferents; given their potential role in the spatial aspects of bodily self‐consciousness (see Pfeiffer et al. 2014 for a review) and the integration between cerebellar and vestibular inputs (McCall et al. 2017), our results could be due, at least in part, to a direct modulation of the vestibular system. Nonetheless, vestibular signals alone are not sufficient, as they are signaling head position, but not the position of other body parts with regards to the extra‐ and peripersonal space; a wide network, ranging from the temporo‐parietal junction (TPJ) to parieto‐occipital and medial‐temporal cortices, is involved in the bodily self‐consciousness within the peripersonal space (Pfeiffer et al. 2014; Blanke et al. 2015).

A further alternative explanation for our results is that the cerebellum could be responsible for the proprioceptive memory of the position of the limb: along this view, HBR is reduced after cerebellar stimulation due to the lack of awareness of the proximity of the limb (Koutsikou et al. 2015). Against this hypothesis, HBR is mediated by brainstem circuits rather than by facilitation of facial motorneurons or by presynaptic disinhibition of primary afferents of the hand (Sambo et al. 2012b).

Finally, peripersonal defensive behavior may be investigated with other protective reflexes, such startle or air puff eyeblink. Noteworthy, HBR amplitude is continuously modified as a function of both the current and predicted hand position, depending of the direction of the movement of the stimulus with respect to the body (Wallwork et al. 2016): therefore, compared with other conditioned responses, the neural circuitry subserving HBR ensures appropriate adjustment of defensive behavior in rapidly changing sensory environment. These features make the HBR particularly useful for the evaluation of different mechanisms underlying defensive peripersonal behavior in humans.

## Conclusions

In this study, we have induced a transient perturbation of cerebellar function to elucidate the role of cerebellum in the peripersonal defensive behavior in humans. Present results suggest that cerebellum is engaged in visual‐independent defensive behavior and integrate previous evidence supporting a critical role of the cerebellum in the genesis, control, and memory of the conditioned eyeblink response (McCormick et al. 1983). In addition, our data seem to indicate that cerebellum is not only involved in the integration of motor and nonmotor functions, but also contributes to disentangle different channels carrying motor and multisensory information.

## Conflict of Interest

None declared.

## Data Accessibility
